# Overlooked components of lipid metabolism: Odd‐chain polyunsaturated fatty acids in photosynthetic microorganisms

**DOI:** 10.1111/jpy.70211

**Published:** 2026-07-27

**Authors:** Linda Nedbalová, Milada Vítová, Tomáš Řezanka

**Affiliations:** ^1^ Department of Ecology, Faculty of Science Charles University Prague Czech Republic; ^2^ Institute of Botany Czech Academy of Sciences Třeboň Czech Republic; ^3^ Institute of Microbiology Czech Academy of Sciences Prague Czech Republic

**Keywords:** algae, cyanobacteria, dinoflagellates, *Euglena gracilis*, fatty acid biosynthesis, GC‐MS, lipidomics, odd chain polyunsaturated fatty acids, propionyl‐CoA

## Abstract

This mini‐review examines odd‐carbon polyunsaturated fatty acids (odd‐PUFAs), a rare but biologically significant group of fatty acids in photosynthetic microorganisms. Odd‐PUFAs are straight‐chain fatty acids containing an odd number of carbon atoms and two or more methylene‐interrupted double bonds. They typically account for less than 1% of total fatty acids in microalgae and cyanobacteria. Current evidence indicates that their biosynthesis primarily originates from propionyl‐CoA‐initiated fatty acid synthesis, followed by conventional elongation and desaturation reactions. The review focuses on three major themes. First, it addresses analytical challenges in odd‐PUFA identification, emphasizing the limitations of gas chromatography with a flame ionization detector (GC‐FID) retention‐time assignments and the need for complementary tandem mass spectrometry (MS/MS)‐based approaches and derivatization techniques for reliable structural characterization. Second, it summarizes the current knowledge of odd‐PUFA biosynthesis, including the metabolic origins of propionyl‐CoA and its incorporation into fatty acid synthase pathways. Third, it surveys the occurrence of odd‐PUFAs across photosynthetic microorganisms, ranging from trace constituents in cyanobacteria and chlorophytes to the characteristic profiles of *Euglena gracilis* and the very‐long‐chain odd‐PUFAs in dinoflagellates. The primary emphasis is on photosynthetic microorganisms, although thraustochytrids are included as a comparative group because they represent the best characterized microbial system for odd‐chain PUFA biosynthesis. Higher trophic organisms are discussed only briefly to illustrate the transfer of odd‐PUFA algal through aquatic food webs. Advances in lipidomics and the growing interest in odd‐chain fatty acids highlight the need for more research, including analytical standardization, broader taxonomic surveys, and metabolic engineering approaches for biotechnological production.

**Abbreviations**
APCI: atmospheric pressure chemical ionization
CL: cardiolipin
DGDG: digalactosyldiacylglycerol
DMOX: dimethyloxazoline
EI‐MS: electron ionization mass spectrometry
ESI: electrospray ionization
even‐PUFA: even‐carbon polyunsaturated fatty acid
FA: fatty acid
FAME: fatty acid methyl ester
FAS: fatty acid synthase
GC‐FID: gas chromatography with flame ionization detection
GC‐MS: gas chromatography–mass spectrometry
LC‐MS: liquid chromatography–mass spectrometry
MS/MS: tandem mass spectrometry
NMI PUFA: non‐methylene interrupted PUFA
odd‐PUFA: odd‐carbon polyunsaturated fatty acid
odd‐VLCPUFA: odd‐chain very‐long‐chain polyunsaturated fatty acid
PKS: polyketide synthase
TAG: triacylglycerol

## INTRODUCTION

In this review, odd‐chain polyunsaturated fatty acids (odd‐PUFAs) are defined as normal, that is, straight‐chain fatty acids with an odd number of carbon atoms and two or more methylene‐interrupted double bonds. This definition excludes branched‐chain fatty acids, regardless of carbon number. Unlike even‐chain PUFAs (even‐PUFAs), odd‐PUFAs are often overlooked but widely distributed due to their relatively low abundance, typically comprising less than 1% of total fatty acids in microorganisms, animals, and humans. They occur less frequently in higher plants, and some reported occurrences require further verification (Diedrich & Henschel, 1990; Řezanka & Sigler, 2009; Schlenk, 1971, 1972). Despite their low abundance, odd‐PUFAs are metabolized through pathways analogous to those of even‐numbered fatty acids.

Growing recognition of the biological roles for saturated odd‐chain fatty acids—particularly pentadecanoic acid (15:0) and heptadecanoic acid (17:0) as biomarkers of dairy consumption and metabolic health indicators (Brevik et al., 2005; Venn‐Watson et al., 2020)—motivates parallel investigation of their polyunsaturated analogs in photosynthetic microorganisms.

Odd‐chain fatty acids are biosynthesized through two primary pathways: (1) α‐oxidation of even‐chain FAs, removing one carbon or (2) the elongation of propionyl‐CoA instead of acetyl‐CoA as a primer. For all unsaturated fatty acids, multiple double‐bond positions are possible, some being considered essential and acting similarly to prostaglandins. Processes such as desaturation, chain elongation, or chain shortening are possible, as observed with even‐numbered PUFAs.

Existing reviews of odd‐chain fatty acids provide limited coverage of polyunsaturated species. Dąbrowski and Konopka (2022) comprehensively reviewed saturated and monounsaturated odd‐chain fatty acids but did not address odd‐PUFAs. Similarly, another review by L. Zhang et al. (2020) on the microbial synthesis of functional odd‐chain fatty acids provided only a limited number of examples of odd‐PUFAs. Notable exceptions included the presence of odd‐PUFAs such as 17:2ω‐5, 17:2ω‐8, and 17:3ω‐5 in chytrid fungi (Akinwole et al., 2014) and the series of C19 and C21 PUFAs in thraustochytrids (Lee Chang et al., 2011).

These scattered reports suggest that odd‐PUFAs are more widespread than generally recognized, warranting a focused review of their occurrence, biosynthesis, and analytical identification in photosynthetic microorganisms. The present review centers on microalgae and cyanobacteria as primary producers. Thraustochytrids—heterotrophic stramenopiles phylogenetically related to diatoms and chrysophytes—have been included as a comparative reference because they represent the most thoroughly characterized microbial system for odd‐chain PUFA biosynthesis and provide a useful context for understanding polyketide‐type PUFA pathways in stramenopile lineages. Organisms at higher trophic levels (fish, marine invertebrates) are discussed briefly as evidence for the trophic transfer of algal odd‐PUFAs.

### Nomenclature

In fatty acid shorthand nomenclature, the number before the colon specifies the number of carbon atoms and that after the colon specifies the number of double bonds. Polyunsaturated fatty acids (PUFAs) are fatty acids with methylene‐interrupted double bonds, that is, with two or more double bonds of the cis/Z configuration (according to Cahn–Ingold–Prelog priority rules for absolute configuration it is correctly denoted as Z, from the German *zusammen*, meaning “together”) separated by a single methylene group. The fatty acids are designated 9Z,12Z‐18:2 or 9Z,12Z,15Z‐18:3, respectively. Biochemists and nutritionists find it useful to denote the position of the terminal double bond in the form (ω‐*x*), where ω indicates the end CH3 of FA and *x* is the number of carbon atoms from the last double bond. In this form, the shorthand notations for the two acids mentioned above, that is, linoleic and alpha‐linolenic acids, are 18:2ω‐6 and 18:3ω‐3.

### Odd‐PUFAs as standards

Several odd‐PUFAs are commercially available as analytical standards: for example, 17:3ω‐3, 19:2ω‐6, 19:3ω‐3, 19:3ω‐6, 19:4ω‐3, 19:5ω‐3, 21:3ω‐3, 21:3ω‐6, 21:4ω‐6, 21:5ω‐3, 23:5ω‐3, 23:6ω‐3, and others. However, C15 odd‐PUFAs are not commercially available according to the SciFinder and Reaxys databases (as of January 2026), and only one C17 PUFA can be purchased. The limited availability of commercial standards necessitates either chemical synthesis or isolation from natural sources for many odd‐PUFA studies.

## SYNTHESIS OF ODD‐PUFAs

Two main strategies exist for the synthesis of odd‐PUFAs. The first involves chain homologation of even‐PUFA precursors by one carbon atom, using reactions such as Grignard carbonation with CO_2_, the Arndt‐Eistert homologation (Larsen et al., 1997), or the Kowalski ester homologation (Organic Chemistry Portal, n.d.). As a specific example, Itoh et al. (2011) synthesized 23:6ω‐3 from docosahexaenoic acid (22:6ω‐3) via chain extension using potassium cyanide (KCN). The second approach is total organic synthesis, in which the odd‐chain starter unit is introduced de novo; acetylenic coupling strategies (e.g., Kunau et al., 1971; Ng et al., 2017) allow systematic access to odd‐chain PUFAs with defined double‐bond positions. Comprehensive reviews discussing various methods of total synthesis are available (Durand et al., 2000; Gunstone, 1957; Sprecher, 1977). Figures illustrating the Kowalski homologation and Arndt‐Eistert reaction schemes are provided as Figures S1 and S2.

## ANALYSIS

### Gas chromatography–mass spectrometry (GC‐MS) identification and its limitations

Modern GC‐MS and liquid chromatography–mass spectrometry (LC‐MS) instrumentation enables reliable detection of odd‐PUFAs, although the low natural abundance and structural similarity of odd‐PUFAs to even‐chain isomers present analytical challenges. For the analysis of derivatives of FAs, such as methyl esters or derivatives containing nitrogen in the molecule (for better interpretation of the position of double bonds), it is sufficient to use GC‐MS. However, positional isomers exhibit remarkably similar mass spectra and overlapping retention times, complicating unambiguous identification. For example, positional isomers of 5,8,12,14‐20:4, 5,8,13,17‐20:4, 5,8,14,16‐20:4, 5,11,14,17‐20:4, 20:4ω‐6, 20:4ω‐3, and 20:4ω‐1 yield nearly identical mass spectra, with differentiation possible only through comparison of retention time when using a capillary column with both polar and non‐polar phases (Christie, 2025). A further complication is the typically low and variable abundance of odd‐PUFAs in natural samples, which is discussed in the relevant sections below.

### Derivatization strategies for double‐bond localization

Unambiguous determination of double‐bond positions in odd‐PUFAs requires derivatization with nitrogen‐containing reagents followed by GC‐MS. The two most widely used approaches are (i) 3‐pyridylcarbinol (formerly picolinyl) esters, which produce characteristic fragment ions reflecting the carbon skeleton and allowing precise double‐bond localization; and (ii) pyrrolidide derivatives, offering complementary fragmentation patterns. Mass spectral reference databases are available for both derivative types (Christie, 2025), providing, for example, spectra of 19:4ω‐5 and 21:5ω‐3 picolinyl esters and the pyrrolidide of 21:5ω‐3. As an original example, we present the previously unpublished electron impact mass spectrum of 3‐pyridylcarbinol 17:3ω‐5 (Figure 1), demonstrating the utility of this approach for odd‐PUFA structure confirmation. 4,4‐Dimethyloxazoline (DMOX) derivatives have also been employed in large‐scale surveys: Lang et al. (2011) used DMOX alongside fatty acid methyl ester (FAME) analysis for the SAG culture collection, and Lee Chang et al. (2011, 2012) used DMOX to confirm odd‐PUFA structures in thraustochytrids, although double‐bond positions remained unassigned for several compounds even with this approach.

**FIGURE 1:**
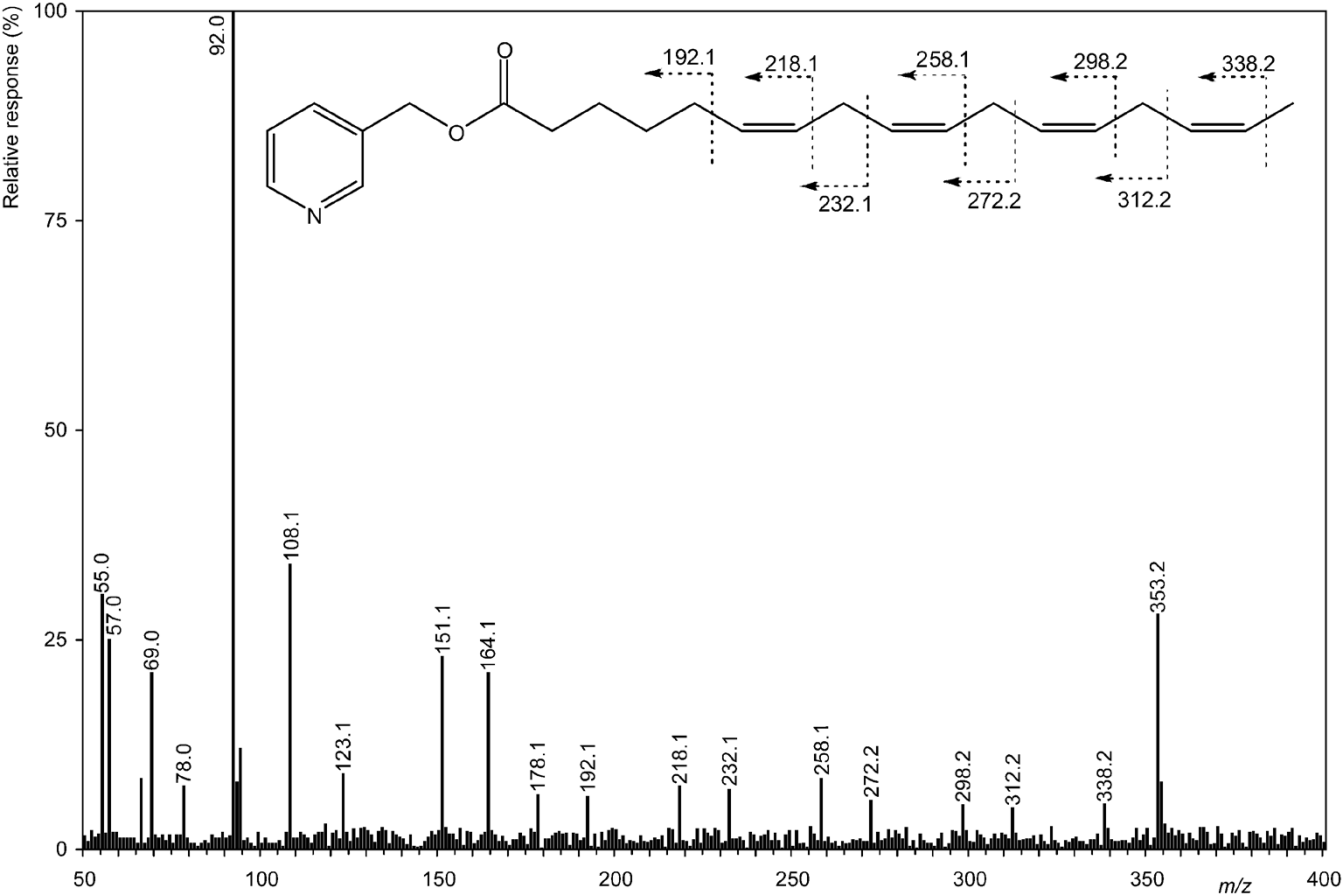
Mass spectrum of 3‐pyridylcarbinol 17:3ω‐5.

### LC‐MS for intact lipid identification

For odd‐PUFAs incorporated into complex lipids—triacylglycerols (TAGs), phospholipids, and glycolipids—transesterification followed by GC‐based methods yields information only on the constituent fatty acids, not on their positional arrangement within the intact lipid. The only applicable method for intact lipid identification is LC‐MS with soft ionization, that is, the atmospheric pressure chemical ionization (APCI) or electrospray ionization (ESI). Both approaches have been used successfully for odd‐PUFA‐containing lipids in photosynthetic microorganisms, including APCI‐LC‐MS characterization of odd‐VLCPUFAs in *Amphidinium carterae* (Řezanka et al., 2008b) and ESI‐based identification of 19:5‐containing cardiolipin (CL) and digalactosyldiacylglycerol (DGDG) in Symbiodiniaceae (La Motta et al., 2024). Dozens of additional analyses are documented in the freely available “Lipids – Literature Surveys” database (Christie, 2026), which serves as a valuable reference resource for method development and spectral interpretation.

## BIOSYNTHESIS

### Metabolic origins of propionyl‐CoA in photosynthetic microorganisms

The biosynthesis of odd‐chain fatty acids begins with the availability of propionyl‐CoA (C3) as an alternative starter unit to the canonical acetyl‐CoA (C2) in fatty acid synthase (FAS) systems. In photosynthetic microorganisms, propionyl‐CoA can arise from several metabolic sources: (i) catabolism of odd‐chain amino acids—isoleucine, valine, and methionine—are degraded via the methylmalonyl‐CoA pathway to yield propionyl‐CoA; (ii) β‐oxidation of odd‐chain fatty acids, which yields one molecule of propionyl‐CoA at the final thiolysis step alongside multiple acetyl‐CoA units; and (iii) fermentative or low‐oxygen‐related pathways in which propionate is produced and subsequently activated to propionyl‐CoA. A competing metabolic fate for propionyl‐CoA is its conversion to succinyl‐CoA via three enzymes—propionyl‐CoA carboxylase, methylmalonyl‐CoA epimerase, and methylmalonyl‐CoA mutase—allowing entry into the tricarboxylic acid cycle. The balance between these competing fates (succinyl‐CoA entry vs. FAS initiation) determines the flux into odd‐chain fatty acid biosynthesis and remains poorly characterized in photosynthetic microorganisms.

### Propionyl‐CoA as a FAS starter unit (Figure 2)

**FIGURE 2:**
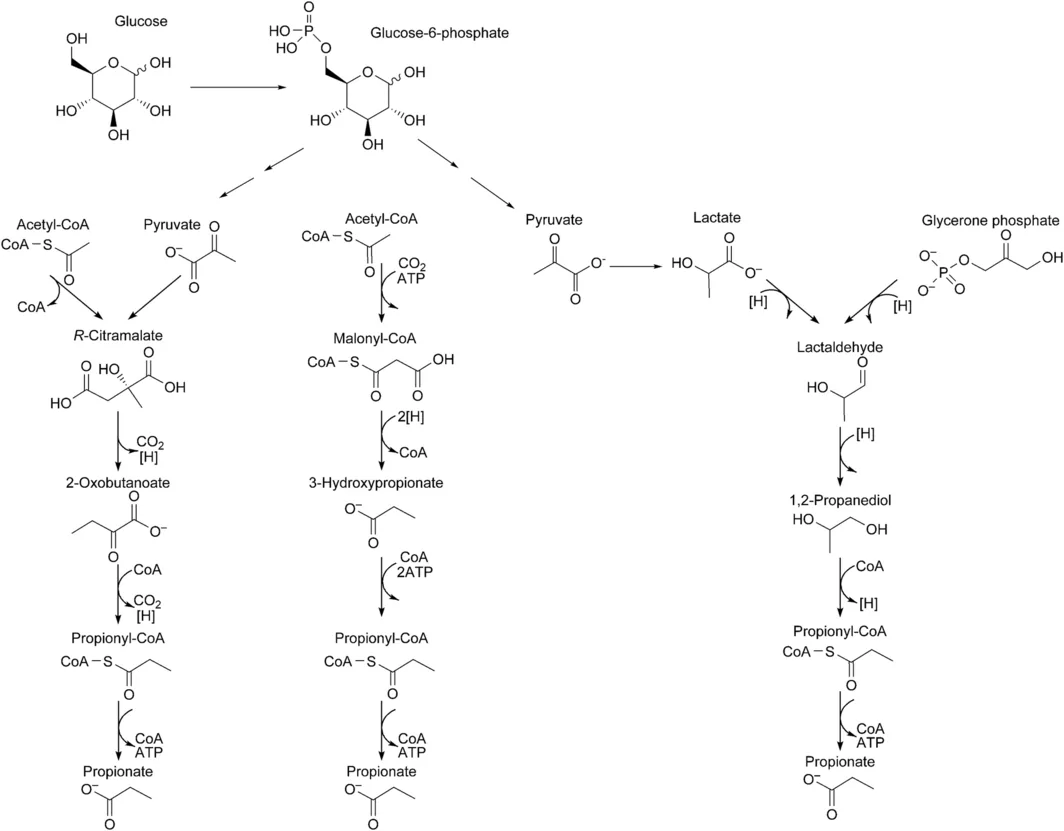
Possible metabolism of propionyl‐CoA is its utilization as a starter unit for the synthesis of odd‐chain fatty acids.

Direct experimental evidence that the FAS machinery in photosynthetic microorganisms can accept propionyl‐CoA as a starter unit comes from precursor‐directed biosynthesis experiments by Beld et al. (2016), who supplemented cultures of *Synechocystis* sp., *Chlamydomonas reinhardtii*, *Chlorella vulgaris*, and *Thalassiosira pseudonana* with short odd‐chain fatty acid precursors (C3:0, C5:0, C9:0). Exogenous propionate and its homologs were successfully incorporated and elongated to C15 and C17 saturated and monounsaturated products in all tested species, demonstrating that FAS substrate specificity is not absolutely restricted to even‐chain primers. However, subsequent desaturation to generate PUFAs was limited: Only *Synechocystis* sp. produced detectable odd‐PUFAs (specifically 17:2), and only in minor amounts, suggesting that although FAS can accept odd‐chain starters, the desaturase step represents a bottleneck in most species tested. An alternative route involving methylmalonyl‐CoA as an extender unit in a polyketide synthase (PKS)‐based pathway—analogous to even‐chain PUFA biosynthesis in thraustochytrids—has not yet been tested in photosynthetic organisms (Ma et al., 2023).

In addition to the classical aerobic biosynthetic pathway for PUFA synthesis in almost all eukaryotes, which involves fatty acid synthase (FAS) followed by the action of elongases and desaturases to introduce double bonds, a polyketide PUFA synthase pathway is used in some prokaryotes (Řezanka et al., 2020) and eukaryotes (François et al., 2025; Remize et al., 2020). Experiments involving the use of methyl malonyl‐CoA (propionate) extender units as starter units in the biosynthesis of odd‐PUFAs via polyketide synthase (PKS) have not yet been performed. The comparative analysis of odd‐PUFA production in model organisms using conventional FAS pathways provides baseline data for future PKS engineering approaches.

### Desaturation and elongation to odd‐PUFAs

Once an odd‐chain saturated or monounsaturated FA is formed via propionyl‐CoA priming, it serves as a substrate for the same desaturase and elongase enzymes responsible for even‐chain PUFA biosynthesis. The most direct experimental evidence comes from Δ4 desaturase inhibitor studies in *Euglena gracilis* and *Desmodesmus quadricauda* using resveratrol and polydatin (Řezanka et al., 2017). In *E. gracilis*, inhibition of Δ4 desaturase caused accumulation of 7,10,13‐15:3 (the Δ4‐deficient precursor of 4,7,10,13‐15:4) and a reciprocal decrease in 15:4, while in even‐chain FAs, the 16:3/16:4 ratio showed a complementary reversal—directly demonstrating that Δ4 desaturase acts on both odd‐ and even‐chain PUFA substrates with comparable efficiency. The α‐oxidation of even‐chain 2‐hydroxy fatty acids represents a secondary, alternative route to odd‐chain fatty acids (Jansen & Wanders, 2006; Qin et al., 2023), but direct evidence for this pathway as a significant source of odd‐PUFAs in photosynthetic microorganisms is currently lacking and this route should be considered speculative pending isotope‐labelling or genetic evidence.

## OCCURRENCE IN NATURE

The occurrence of odd‐PUFAs in nature is almost certainly underreported for two convergent reasons. First, most lipidomic surveys of microalgae and cyanobacteria have not specifically targeted minor odd‐chain components; their low abundance (typically <1% of total FAs) places them near or below gas chromatography with flame ionization detection (GC‐FID) limits in routine profiling experiments. Second, even when detected, the structural identity of odd‐chain components is often ambiguous because most reports rely on GC‐FID retention‐time identification without the derivatization‐MS confirmation required for rigorous positional assignment, as discussed in the Analysis section.

The most comprehensive survey of odd‐PUFA occurrence across photosynthetic microorganisms to date is the analysis of fatty acid profiles from more than 2000 strains in the SAG culture collection by Lang et al. (2011), using GC‐MS of FAMEs and DMOX derivatives. This survey identified odd‐PUFAs in cyanobacteria (*Nostoc*, *Synechocystis*), green algae (*Haematococcus*, *Lobomonas*, *Mantoniella*), and Euglenophyceae (*Colacium*, *Euglena*), demonstrating that odd‐PUFAs are more widespread among photosynthetic microorganisms than generally recognized. Notably, double‐bond positions remained uncharacterized for the majority of detected odd‐PUFAs in that survey, underscoring the analytical gap discussed above.

An effective strategy for overcoming low‐abundance detection limits is precursor‐directed biosynthesis via laboratory cultivation: Supplementing growth medium with exogenous odd‐chain precursors (propionate, valerate) stimulates incorporation and elongation to detectable levels, enabling structural characterization that would be impractical from natural abundance material. In our previous work, this approach enabled production of quantifiable odd‐PUFA amounts in *Euglena gracilis* and *Desmodesmus quadricauda* (Řezanka et al., 2015, 2017), facilitating the desaturase inhibitor studies described in the Biosynthesis section. The consistent detection of odd‐PUFAs across trophic levels—from diatoms through to zooplankton, fish, and marine mammals—with 21:5ω‐3 as the most frequently reported species (0.1%–1.1% of total FAs) confirms that algal odd‐PUFAs are transferred through food webs (Mayzaud & Ackman, 1978); these higher trophic‐level data are discussed in the Other Organisms section.

## PHOTOSYNTHESIZING MICROORGANISMS

Photosynthetic microorganisms span an enormous phylogenetic range, from prokaryotic cyanobacteria to numerous eukaryotic lineages that acquired photosynthesis through primary or secondary endosymbiosis. This diversity is paralleled by metabolic diversity in fatty acid composition, making photosynthetic microorganisms a particularly rich yet underexplored source of unusual lipids, including odd‐PUFAs. The following subsections survey documented odd‐PUFA occurrences by taxonomic group, highlighting the analytical methods used for confirmation and the biological context were known.

### Cyanobacteria

Among prokaryotic phototrophs, odd‐PUFAs have been detected in cyanobacteria of the genera *Nostoc* and *Synechocystis* in the SAG culture collection survey (Lang et al., 2011), although double‐bond positions were not fully characterized in that study. More direct experimental evidence comes from Beld et al. (2016), who demonstrated that *Synechocystis* sp. produces detectable 17:2 when supplemented with short odd‐chain precursors—the only photosynthetic organism in their survey to generate an odd‐PUFA under these conditions, suggesting some endogenous desaturase activity toward odd‐chain substrates. The biosynthetic capacity of cyanobacteria for odd‐PUFAs under natural conditions remains poorly characterized and warrants systematic investigation.

### Chlorophytes

Green microalgae constitute one of the best‐documented groups for odd‐PUFA occurrence. The snow alga *Chloromonas brevispina* (Chlamydomonadales), collected from green snowfield patches in the Bohemian Forest, Czech Republic, was found to contain odd‐PUFAs comprising approximately 3% of total FAs, with structures rigorously confirmed by GC‐MS of 3‐pyridylcarbinol ester derivatives (Řezanka et al., 2008a; Table 1). Six odd‐chain di‐ and tri‐unsaturated FAs were identified, including 6,9‐15:2, 6,9,12‐15:3, 5,9‐17:2, 6,9‐17:2, 9,12‐17:2, and 6,9,12‐17:3, all in the range 0.12%–0.53% of total FAs. The SAG survey also identified odd‐PUFAs in *Haematococcus*, *Lobomonas*, and *Mantoniella* (Lang et al., 2011). Precursor incorporation experiments with *Chlamydomonas reinhardtii* and *Chlorella vulgaris* (Beld et al., 2016) confirmed that chlorophyte FAS can accept odd‐chain starters, though subsequent desaturation was limited to saturated and monoenoic products in these species.

**TABLE 1:** Fatty acid composition from the snow alga *Chloromonas brevispina* as determined by GC‐MS (Řezanka et al., 2008a).

Fatty acid	ω abbreviations	%^a^
5,8‐11:2	(ω‐3)	0.59
3,6‐12:2	(ω‐6)	0.71
6,9‐12:2	(ω‐3)	0.83
3,6,9‐12:3	(ω‐3)	0.48
**4,7‐13:2** ^b^	**(ω‐6)**	0.12
**4,7,10‐13:3**	**(ω‐3)**	**0.30**
8‐14:1	(ω‐6)	2.38
5,8‐14:2	(ω‐6)	2.91
5,8,11‐14:3	(ω‐3)	4.63
**6,9‐15:2**	**(ω‐6)**	**0.36**
**6,9,12‐15:3**	**(ω‐3)**	**1.37**
16:0		2.49
16:1	(ω‐9)	8.91
16:1	(ω‐7)	2.97
3t‐16:1^c^	(ω‐13)	0.89
5,9‐16:2		2.02
7,10‐16:2	(ω‐6)	2.91
9,12‐16:2	(ω‐4)	2.14
4,7,10‐16:3	(ω‐6)	5.76
6,9,12‐16:3	(ω‐4)	3.98
7,10,13‐16:3	(ω‐3)	3.15
9,12,15‐16:3	(ω‐1)	2.67
4,7,10,13‐16:4	(ω‐3)	4.69
6,9,12,15‐16:4	(ω‐1)	4.87
9‐17:1	(ω‐8)	1.19
**5,9‐17:2**		**0.12**
**6,9‐17:2**	**(ω‐8)**	**0.36**
**9,12‐17:2**	**(ω‐5)**	**0.42**
**6,9,12‐17:3**	**(ω‐5)**	**0.53**
**8,11,14‐17:3**	**(ω‐3)**	**0.27**
9‐18:1	(ω‐9)	7.13
5,9‐18:2		0.97
6,9‐18:2	(ω‐9)	1.28
9,12‐18:2	(ω‐6)	5.11
12,15‐18:2	(ω‐3)	1.49
5,9,12‐18:3		2.05
6,9,12‐18:3	(ω‐6)	4.06
9,12,15‐18:3	(ω‐3)	6.16
11,14,17‐18:3	(ω‐1)	0.75
5,9,12,15‐18:4		0.81
6,9,12,15‐18:4	(ω‐3)	2.18
8,11,14,17‐18:4	(ω‐1)	1.70
3,6,9,12,15‐18:5	(ω‐3)	1.29

^a^ Percent of total FAs.

^b^ Bold—odd‐chain PUFAs.

^c^ Trans (*E*) configuration of double bonds.

### Euglenophytes

The freshwater euglenid *Euglena gracilis* has emerged as the best‐characterized photosynthetic microorganism for odd‐PUFA metabolism, owing to its diverse odd‐PUFA profile and tractable cultivation. Two analyses conducted more than 50 years apart demonstrated remarkable consistency. Korn (1964) identified 51 fatty acids using mercuric acetate fractionation and GC‐FID with permanganate‐periodate oxidation for double‐bond assignment, among them six diunsaturated odd‐PUFAs (C15–C19), one triunsaturated odd‐PUFA (17:3ω‐2), and the polyunsaturated odd‐PUFAs 15:4ω‐2, 19:4ω‐5, 21:4ω‐5, and 21:5ω‐3. Re‐analysis by Řezanka et al. (2015) using modern GC‐MS on highly polar ionic‐liquid capillary columns yielded a strikingly similar odd‐PUFA profile (Table 2), demonstrating that odd‐PUFA biosynthetic capacity is a stable phenotypic trait in wild‐type *E. gracilis* strains.

**TABLE 2:** Odd‐PUFAs of *Euglena gracilis*.

FA	% of total FA^a^	% of total FA^b^
15:2ω‐8	0.29	0.2
15:2ω‐5	0.58	0.8
15:3^c^	0.05	tr
15:4ω‐2	0.70	0.6
17:2ω‐7	0.70	0.5
17:2ω‐5	0.23	1.5
17:3ω‐2	1.75	1.5
17:4^c^	0.12	tr
19:2ω‐7	0.12	0.7
19:2ω‐5	0.23	1.3
19:3^c^	0.05	tr
19:4ω‐5	0.70	6.0
21:4ω‐5	0.35	1.0
21:5ω‐5	0.00	1.5

^a^ Řezanka et al. (2017).

^b^ Korn (1964).

^c^ Double bond position undetermined.

The biosynthetic pathway was probed using selective Δ4 desaturase inhibitors (resveratrol and polydatin; Řezanka et al., 2017). Inhibitor treatment caused accumulation of 7,10,13‐15:3 and a reciprocal decrease in 4,7,10,13‐15:4, whereas in even‐chain FAs, the 16:3/16:4 ratio showed a complementary reversal—directly demonstrating that Δ4 desaturase acts on both odd‐ and even‐chain PUFA substrates with comparable efficiency. *Euglena gracilis* thus represents an excellent model for future metabolic engineering and isotope‐labelling studies of odd‐PUFA biosynthesis in photosynthetic microorganisms. The colorless euglenoid *Khawkinea quartana* also incorporates odd‐PUFAs (notably 19:4) into triacylglycerols; all three regioisomers were resolved by coupled reverse‐phase and chiral‐phase LC‐MS (Řezanka et al., 2015), illustrating the power of modern chromatographic approaches for complete structural characterization of odd‐PUFA‐containing complex lipids.

### Diatoms and other stramenopiles

Among diatoms, *Skeletonema costatum* holds the distinction of being among the first photosynthetic organisms in which an odd‐PUFA (21:5) was documented, more than 60 years ago (Ackman et al., 1964). Precursor incorporation experiments with the diatom *Thalassiosira pseudonana* (Beld et al., 2016) confirmed FAS‐mediated incorporation of odd‐chain starters, although without PUFA formation in that species. The chrysophyte *Ochromonas danica* provided a particularly clear demonstration of odd‐PUFA metabolic capacity: Endogenous odd‐PUFAs were present at only 0.01%–0.15% of total FAs, but when 17:3ω‐5 was added exogenously, it was elongated and desaturated to yield 19:3ω‐5 (up to 12.6%) and 19:4ω‐5 (up to 4.2%), demonstrating robust metabolic capacity that is substrate‐limited rather than enzyme‐limited (Gellerman & Schlenk, 1972).

### Haptophytes

Haptophytes such as *Gephyrocapsa huxleyi* (formerly *Emiliania huxleyi*) have received extensive attention as sources of ω‐3 PUFAs, although studies have focused almost exclusively on even‐chain species. *Nannochloropsis* sp. was reported to contain 21:5ω‐3 by GC‐FID using a 37‐component FAME standard mix (Valente et al., 2019); however, since this standard mixture does not include 21:5ω‐3, the structural assignment must be considered tentative—a cautionary example of the identification limitations discussed in the Analysis section.

### Dinoflagellates

Dinoflagellates are distinguished among photosynthetic microorganisms by their capacity to produce odd‐numbered very‐long‐chain PUFAs (odd‐VLCPUFAs, defined as >21 carbons). An APCI‐LC‐MS analysis of *Amphidinium carterae* identified more than 20 distinct odd‐VLCPUFAs ranging from 21:5ω‐3 to 33:6ω‐3 (Řezanka et al., 2008b), demonstrating extensive chain elongation capability unique to this group. A lipidomic analysis of cultured Symbiodiniaceae—*Breviolum psygmophilum*, *Cladocopium goreaui*, and *Durusdinium trenchii*—revealed three unusual lipid species containing 19:5 PUFA: 15:0/24:0/16:1/19:5‐CL, O‐20:1/19:5/19:5‐TAG, and 19:5/20:5‐DGDG (La Motta et al., 2024). These complex lipids demonstrate that odd‐VLCPUFAs are not merely present as free fatty acids but are incorporated into diverse glycolipid and phospholipid classes, suggesting functional roles in membrane structure and cellular metabolism. The high proportion of odd‐VLCPUFAs in dinoflagellates compared to other algal groups may reflect their unique lipid metabolism and phylogenetic position, warranting further investigation of the biosynthetic machinery responsible for these extended polyunsaturated chains.

## ODD‐CHAIN HYDROCARBONS DERIVED FROM ODD‐PUFAs

Odd‐numbered hydrocarbons represent an important class of lipid‐derived metabolites that result from decarboxylation of odd‐chain fatty acids. Similar to their fatty acid precursors, these compounds can be biosynthesized through decarboxylation of even‐chain PUFAs, yielding odd‐numbered hydrocarbons with two or more double bonds. Although less extensively studied than their corresponding fatty acids, these hydrocarbons provide insight into lipid metabolism and have taxonomic significance in certain algal groups. The colonial green alga *Botryococcus braunii* produces several odd‐chain polyunsaturated dienes, including heptacosa‐1,18‐diene (C27:2), nonacosa‐1,20‐diene (C29:2), and hentriaconta‐4,22‐diene (C31:2; Knights et al., 1970). Lee and Loeblich (1971) documented the widespread distribution of 21:6 hydrocarbon alongside its presumed 22:6 fatty acid precursor across diverse algal taxa in both marine and freshwater environments, including *Eutreptia viridis*, *Exuviaella cassubica*, *Cryptomonas ovata*, and *Skeletonema costatum*.

As another example, the marine diatom *Rhizosolenia setigera* produces a particularly diverse array of polyunsaturated odd‐chain hydrocarbons (Damsté et al., 2000). These include the commonly occurring n‐C21:6 (all‐(Z) heneicosa‐3,6,9,12,15,18‐hexaene) and several novel heptaenes: all‐(Z) pentacosa‐3,6,9,12,15,18,23‐heptaene; all‐(Z) pentacosa‐3,6,9,12,15,18,24‐heptaene; all‐(Z) heptacosa‐3,6,9,12,15,18,26‐heptaene; and all‐(Z) heptacosa‐3,6,9,12,15,18,25‐heptaene. The authors hypothesized that these heptaenes are biosynthesized through chain elongation of 22:3ω‐3 by two or three C2 units followed by decarboxylation, representing a clear example of the metabolic pathway from even‐chain PUFAs to odd‐chain hydrocarbons. Similarly, *Nannochloropsis salina* contains free mono‐ and polyunsaturated n‐alkenes with odd carbon numbers (Gelin et al., 1997).

Beyond simple hydrocarbons, the benthic haptophyte *Chrysotila lamellosa* produces an array of very‐long‐chain odd‐numbered compounds, including both hydrocarbons (C29:2, C31:2, C31:3, C33:2, and C33:3) and related ketones such as methyl and ethyl alkenones (MeC37:4, MeC39:3, EtC39:3; Rontani et al., 2004). The structural diversity of these compounds suggests complex lipid metabolism in this organism.

The occurrence of odd‐chain hydrocarbons parallels that of very‐long‐chain odd‐PUFAs. Marine dinoflagellates, which are known to produce very‐long‐chain PUFAs such as 28:8ω‐3 and 28:7ω‐6 (Mansour et al., 1999), likely also generate corresponding odd‐chain hydrocarbons through decarboxylation, though comprehensive characterization of these products remains to be performed.

## THRAUSTOCHYTRIDS

Thraustochytrids are unicellular saprotrophic eukaryotes (Labyrinthulomycetes, Stramenopiles) that, while non‐photosynthetic, are phylogenetically related to photosynthetic stramenopiles (diatoms, chrysophytes) and are included here as a comparative reference for understanding polyketide‐type PUFA pathways potentially relevant to their photosynthetic relatives.

Lee Chang et al. (2011) provided the first comprehensive characterization of odd‐chain PUFAs in thraustochytrids, analyzing wild strains from Tasmanian (temperate) and Queensland (tropical) waters using GC‐MS of DMOX derivatives. A total of 11 odd‐PUFAs were identified including positional isomers of C19 and C21 species, at approximately 0.1%–3% of total FAs—roughly two orders of magnitude below their even‐chain counterparts (e.g., 22:6ω‐3 at 26.4%). The authors proposed a biosynthetic scheme using 15:0 as the starter unit, followed by the same elongation/desaturation scheme as for even‐numbered PUFAs. A subsequent survey (Lee Chang et al., 2012) identified 36 odd‐PUFAs across 19 strains, with odd‐PUFAs predominantly in temperate‐water isolates (*Schizochytrium*, *Thraustochytrium*) and significantly lower levels in tropical strains, suggesting temperature‐dependent regulation (Table 3). This pattern was reinforced by Ponnambalan et al. (2025), who detected only saturated odd‐chain FAs (15:0 and 17:0)—no odd‐PUFAs—in thraustochytrids from a Malaysian tropical mangrove ecosystem.

**TABLE 3:** Fatty acid composition (as percentage of total FA) for thraustochytrid strains from southeast Tasmania (Lee Chang et al., 2012).

Odd‐PUFA	CS number 979^a^	CS number 980	CS number 981
19:2ω‐7	0.5	0.3	0.4
19:3ω‐8	0.2	0.1	0.1
19:3ω‐5	0.3	0.3	0.3
19:4ω‐5	2.8	1.5	1.9
21:4ω‐7	2.9	1.8	2.0
21:4ω‐5	0.2	0.2	0.2
21:5ω‐5	0.3	0.1	0.1
21:6ω‐2	0.5	0.4	0.4
23:5ω‐6	0.2	0.1	0.1

^a^ CS number is assigned for strains held in the Australian National Algae Culture Collection.

From a biosynthetic engineering perspective, Wang et al. (2019) demonstrated that heterologous gene expression and ELO3 elongase overexpression in *Schizochytrium* sp. led to a 3.3‐fold increase in odd‐chain saturated FAs (15:0 and 17:0) alongside enhanced docosahexaenoic acid (DHA) production, establishing proof of concept that manipulating odd‐chain starter‐unit availability can shift the FAS product profile—a strategy potentially applicable to odd‐PUFA enhancement in photosynthetic stramenopile relatives.

## OTHER ORGANISMS

Odd‐PUFAs identified in organisms at higher trophic levels document the trophic transfer of algal odd‐PUFAs through marine food webs rather than de novo production and provide confirmation of their ultimate algal origin. A key paper by Mayzaud and Ackman (1978) documented the presence of 21:5ω‐3 across trophic levels, from the diatom *Skeletonema costatum* through marine invertebrates, fish, and mammals. This odd‐PUFA consistently appeared as a minor lipid component, ranging from 0.1% to 1.1% of total FAs, with structure unambiguously confirmed by GC‐MS of pyrrolidide derivatives. The Cayman Chemical Company states in its catalog that 6Z,9Z,12Z,15Z,18Z‐heneicosapentaenoic (21:5ω‐3) fatty acid is present in trace amounts in the green alga *Bryopsis pennata* and in fish oils, although without providing specific references.

As early as 1967, Ackman et al. (1967) analyzed methyl esters of FAs derived from cod liver oil (*Gadus morhua*) using capillary columns with a polar stationary phase (butanediolsuccinate polyester). In addition to even‐PUFAs, two odd‐PUFAs, 21:5ω‐2 and 21:6ω‐2, were identified at levels that represent approximately one‐tenth of a percent of total FAs. This pioneering analytical work demonstrated both the detectability of odd‐PUFAs and their presence in commercially important species.

One of the richest sources of odd‐PUFAs is mullet oil (*Mugil cephalus*), which contains more than 25% straight‐chain odd‐PUFAs (Sen & Schlenk, 1964). This fish inhabits coastal waters in tropical, subtropical, and temperate zones around the world. A total of 11 odd‐PUFAs were identified, the most abundant being 17:2ω‐5 and 19:4ω‐5 (Table 4). In contrast, many other species of fish contain 21:5ω‐3 as the major odd‐PUFA. For example, this acid was detected in seal oil (Mayzaud & Ackman, 1978) and in smelt (*Osmerus mordax*; Addison & Ackman, 1970) and many other fish, for example, cod (*Gadus* spp.), saithe (*Pollachius virens*), monkfish (*Squatina squatina*), orange roughy (*Hoplostethus atlanticus*), menhaden (*Brevoortia* spp.) (McGill & Moffat, 1992), and eels (*Anguilla japonica*; Yamamoto et al., 1991).

**TABLE 4:** Odd‐PUFAs in mullet (*Mugil cephalus*; Sen & Schlenk, 1964).

Fatty acid	% of total FA
15:2ω‐6	0.05
15:2ω‐3	0.1
15:3ω‐3	0.2
17:2ω‐7	0.2
17:2ω‐5	2.5
17:3ω‐5	1.2
17:4ω‐2	0.5
19:2ω‐7	0.08
19:2ω‐6	0.6
19:2ω‐5	0.1
19:4ω‐5	1.7

Freshwater fish have shown similar patterns. Four species—brown trout (*Salmo trutta*), channel catfish (*Ictalurus punctatus*), black bullhead (*Ictalurus melas*), and largemouth bass (*Micropterus salmoides*) all contained 21:5ω‐3 as the sole odd‐PUFA at concentrations up to 1% of total FAs (Simonetti et al., 2008).

Marine invertebrates exhibit greater odd‐PUFA diversity. The coral *Lobophyllia corymbosa* (Red Sea, East Africa, Indo‐Pacific) shows unusually high odd‐PUFAs (possibly from symbiotic dinoflagellates), for example, 19:3 (13.9% of total FA), as determined using an SP‐2310 column (Al‐Lihaibi et al., 1998). The sea slug *Scaphander lignarius* contains heneicosa‐5,8,11,14‐tetraenoic acid (21:4ω‐7) at 9.22% (Vasskog et al., 2012), and the amphipod *Pontoporeia femorata* contains multiple odd PUFAs (C15‐C21) across all life‐cycle stages (Paradis & Ackman, 1976). Several invertebrates produce non‐methylene‐interrupted (NMI) odd‐PUFAs with unusual double bond spacing. The ovaries of limpet *Cellana toreuma* contain novel terminal odd non‐methylene interrupted PUFA (NMI PUFAs; e.g., 7,18‐19:2, 11,20‐21:2), identified by GC‐MS/EI‐MS and synthesis (Kawashima, 2018; Kawashima & Ohnishi, 2017). The deep‐sea clam *Calyptogena phaseoliformis* contains NMI‐PUFAs (7,16‐21:2; 4,7,16‐21:3), contrasting with shallow‐water *Mactra chinensis* with 21:5ω‐3 (Saito, 2007).

The consistent presence of 21:5ω‐3 across diverse consumers confirms algae as the primary source. However, limited bioaccumulation at higher trophic levels suggests preferential metabolism of odd‐chain lipids compared to even‐chain PUFAs.

## CONCLUSIONS

Odd‐chain polyunsaturated fatty acids, although typically below 1% of total fatty acids, are distributed across a wide phylogenetic range of photosynthetic microorganisms—from cyanobacteria and chlorophytes through euglenophytes, diatoms, haptophytes, and dinoflagellates. The available experimental evidence consistently supports propionyl‐CoA‐initiated fatty acid synthesis as the primary biosynthetic route, followed by the same elongation and desaturation machinery responsible for even‐chain PUFA formation. *Euglena gracilis* stands out as the best characterized photosynthetic model for odd‐PUFA metabolism, with stable profiles documented across five decades of analysis and direct enzymatic evidence from inhibitor experiments. Dinoflagellates are remarkable for their capacity to produce odd‐numbered very‐long‐chain PUFAs incorporated into complex glycolipids and phospholipids, pointing to PKS‐related biosynthetic capacity unique to this group.

Three principal challenges currently limit progress in this field. First, analytical identification of odd‐PUFAs remains problematic: Most published occurrence reports rely on GC‐FID retention‐time identification alone, which is insufficient for unambiguous positional assignment, and the commercial unavailability of authentic standards for most C15 odd‐PUFAs and positional isomers constrains rigorous structural confirmation. Second, the biosynthetic pathways are poorly characterized at the molecular level: Propionyl‐CoA metabolism has been directly investigated in only a handful of photosynthetic species, FAS substrate specificity for odd‐chain primers remains incompletely understood, and the relative contributions of FAS versus PKS routes to odd‐PUFA formation are unknown in all photosynthetic taxa. Third, quantitative data across algal taxa are scarce: No systematic lipidomic survey using modern untargeted approaches has specifically targeted these compounds across a broad phylogenetic range.

Future research should prioritize: (1) analytical validation using derivatization‐MS/MS approaches (picolinyl esters, pyrrolidides, DMOX) and synthesis of authentic standards for understudied structures, particularly C15 odd‐PUFAs; (2) isotope labelling with ^13^C‐propionate to trace propionyl‐CoA incorporation and quantify biosynthetic flux in model photosynthetic microorganisms; (3) expanded taxonomic surveys using untargeted lipidomics with sufficient sensitivity to detect minor components; (4) molecular characterization of FAS and PKS substrate specificity in photosynthetic taxa; and (5) metabolic engineering of propionyl‐CoA availability as a strategy to enhance odd‐PUFA titers in biotechnologically relevant algal strains. The identification of odd‐chain PUFA‐containing TAGs as candidate biomarkers for colon cancer profiling (Zhang et al., 2023) provides additional motivation for improving the analytical and biosynthetic foundations of this field.

## AUTHOR CONTRIBUTIONS

**Linda Nedbalová:** Conceptualization (equal); investigation (equal); writing – original draft (equal); writing – review and editing (equal). **Milada Vítová:** Conceptualization (equal); investigation (equal); writing – original draft (equal); writing – review and editing (equal). **Tomáš Řezanka:** Conceptualization (equal); investigation (equal); writing – original draft (equal); writing – review and editing (equal).

## FUNDING INFORMATION

This study was supported by the grant project GA24‐10019S, by the Institutional Research Concepts RVO61388971 (Institute of Microbiology, Czech Academy of Sciences) and RVO67985939 (Institute of Botany, Czech Academy of Sciences), and Grant Talking Microbes—Understanding Microbial Interactions within One Health Framework (CZ.02.01.01/00/22_008/0004597).

## DECLARATION OF GENERATIVE AI AND AI‐ASSISTED TECHNOLOGIES IN THE WRITING PROCESS

During the preparation of this work, the author(s) used ChatGPT in order to improve English language and style. After using this tool/service, the author(s) reviewed and edited the content as needed and take(s) full responsibility for the content of the publication.

## Supporting information


**Figure S1.** Kowalski ester homologation (Organic Chemistry Portal, https://www.organic‐chemistry.org).
**Figure S2**. Arndt‐Eistert reaction.
