# Supplementary material for: Overlooked components of lipid metabolism: Odd‐chain polyunsaturated fatty acids in photosynthetic microorganisms
Source: J Phycol. 2026 Jul 27;62(5):1428–39. doi: 10.1111/jpy.70211 (PMC13659350; doi:10.1111/jpy.70211)
Supplement: Supplementary file 1 [file JPY-62-1428-s001.docx]

Fig. S1. Kowalski ester homologation (Organic Chemistry Portal, <https://www.organic-chemistry.org>)

Fig. S2. Arndt-Eistert reaction.
